# Extracellular vesicles and miRNA-based therapies in triple-negative breast cancer: advances and clinical perspectives

**DOI:** 10.20517/evcna.2024.85

**Published:** 2025-02-14

**Authors:** Caroline Patini de Rezende, Débora de Lima Alves, Luiz Gustavo de Almeida Chuffa, Debora Aparecida Pires de Campos Zuccari

**Affiliations:** ^1^Department of Molecular Biology, Cancer Molecular Research Laboratory (LIMC)/FAMERP, São José do Rio Preto 15090-000, Brazil.; ^2^Department of Anatomy-IBB/UNESP, Institute of Biosciences of Botucatu, Univ.Estadual Paulista, Botucatu 18618-689, Brazil.

**Keywords:** Extracellular vesicles (EVs), miRNAs, EVs-miRNAs therapies, mesenchymal stem cells (MSCs), triple-negative breast cancer (TNBC)

## Abstract

Triple-negative breast cancer (TNBC) is one of the most aggressive and challenging subtypes for treatment, due to the lack of hormone receptors and the human epidermal growth factor receptor 2 (HER2) protein. The identification of new molecular targets is important for the development of targeted and specific therapies for TNBC patients. MicroRNAs (miRNAs) have emerged as promising molecular targets, being involved in cellular processes such as cell survival, apoptosis, differentiation, carcinogenesis, and metastasis. Extracellular vesicles (EVs) have gained prominence in areas such as drug delivery, immune modulation, biomarkers for diagnosis and prognosis, and therapeutics, due to their use as vehicles for the delivery of miRNAs, regulation of gene expression, and development of combined therapeutic strategies. In particular, mesenchymal stem cell-derived EVs (MSC-derived EVs) can transfer proteins, mRNAs/miRNAs, or DNA molecules and are being considered safer treatment options due to their inability to directly form tumors and contain lower amounts of membrane proteins such as MHC molecules. Numerous studies have highlighted the role of miRNAs in EVs in TNBC tumorigenesis, with a focus on diagnosis, prognosis, treatment selection, and monitoring. However, the development of therapies with EVs, especially MSC-derived EVs, is still in its infancy. Therefore, the aim of this review is to address new therapeutic strategies based on the delivery of miRNAs through EVs, with a focus on MSC-derived EVs, for the treatment of TNBC as an innovative therapy in oncology.

## INTRODUCTION

The American Cancer Society (ACS) has estimated cancer incidence and mortality cases in the United States for 2024^[1]^. Approximately 2,001,140 new cases are projected, of which 313,510 are related to female-prevalent breast cancer (BC). Between 2015 and 2019, BC incidence rates increased by 0.6% to 1% annually. However, mortality rates have continued to decline, with an estimated 611,720 deaths in 2024, including 42,780 specific to BC. Survival rates for BC increased by 16% between 1975 and 2019. The decline in mortality - reached 1% annually from 2013 to 2021 - can be attributed to earlier diagnosis, increased awareness, and the development of improved treatment options, as well as new innovative therapies^[1]^. BC is one of the most prevalent neoplasms and its subtypes are classified according to the molecular profiles^[2]^.

The triple-negative breast cancer subtype (TNBC) is characterized by a deficiency in human epidermal growth factor receptor 2 (HER2), and less than 1% expression of estrogen receptors (ER) and progesterone receptors (PR)^[3]^. It represents a specific and aggressive form of BC with invasive behavior, rapid progression, and a lower survival rate^[3]^. Despite remarkable advancements, metastases are responsible for approximately 90% of BC-related fatalities due to therapy-resistant cells^[4]^. Leone *et al*. (2023) estimated the mortality risk in women, aged between 40 and 79 years, with non-metastatic, high-grade TNBC, using the “ESTIMATE-TN” tool. The results showed that, after three years of diagnosis, there was a decrease in the risk of mortality due to TNBC from 30.8% to 17.4%, as well as differences in mortality risks according to age and clinicopathological characteristics of the tumor^[5]^. These findings highlight the need for further investigations to improve prognosis and enable early diagnosis^[5]^.

Cancer therapies reached an important milestone in the 1970s with the clinical study of tamoxifen, the first therapeutic and non-cytotoxic molecule developed for the treatment of BC^[6]^. Currently, cancer treatment methods continue to include chemotherapy, radiotherapy, and surgery; however, these approaches face challenges such as off-target toxicity and limited drug delivery to tumors^[4]^. The discovery of targeted therapies has promoted better efficacy in cancer treatment, promoting increased remission rates, reduced toxicity, and lower mortality^[6]^. Furthermore, the efficacy of TNBC treatment is limited due to the lack of specific molecular targets, so searching for innovative therapeutic approaches is essential^[7]^.

In this perspective, extracellular vesicles (EVs) have been studied as therapeutic delivery channels for anticancer strategies^[8]^, tissue regeneration^[9]^, and vaccination^[10]^. Research on EVs has been growing significantly, addressing diagnostic, prognostic, and therapeutic potential^[11]^. EVs have a diversity of biological functions and transport bioactive molecules, such as proteins, lipids, and nucleic acids, with an emphasis on microRNAs (miRNAs)^[12]^. miRNAs are small non-coding ribonucleic acid (RNA) responsible for the post-transcriptional regulation of gene expression. Studies have shown their actions in several types of cancer, acting as tumor suppressors and inhibiting pro-tumor signaling pathways^[13]^. Direct exposure of BC cells to miRNAs is ineffective for therapeutic purposes^[14]^. miRNAs encapsulated in EVs can be protected against enzymatic degradation, increasing their stability and circulation time, offering an innovative strategy to modulate biological processes^[12]^.

The tumorigenic and pro-metastatic capacity of BC has been associated with the dysregulation of the cellular miRNA profile, which favors the expression of tumor-promoting miRNAs (oncomiRs) and negatively regulates tumor-suppressor miRNAs. This cellular miRNA dysregulation is associated with increased tumor angiogenesis, epithelial-mesenchymal transition (EMT), and adipose tissue remodeling, which favor tumor growth, progression, and metastasis^[15]^. However, the transport and delivery of miRNAs contained in EVs and their roles in promoting tumorigenic and pro-metastatic capacity still need to be fully elucidated. We aim to address new therapeutic strategies based on the delivery of miRNAs through EVs, with a focus on EVs from mesenchymal stem cells (MSCs), for the treatment of TNBC as an innovative therapy in oncology.

In this review, we conducted a comprehensive literature search to identify relevant studies on the role of miRNAs and EVs, particularly MSCs-derived EVs, in TNBC. The references were collected from databases including PubMed, Scopus, and Web of Science, focusing on studies published between 2010 and 2025. The inclusion criteria were studies addressing miRNA delivery via EVs, their therapeutic potential, and specific applications in TNBC. Overlapping studies or reviews with similar content were carefully evaluated to avoid redundancy and ensure the inclusion of novel and relevant findings. This methodological approach ensured a robust foundation for discussing emerging therapeutic strategies based on miRNAs and EVs in TNBC.

## BC: MOLECULAR FOUNDATIONS AND EPIDEMIOLOGICAL ASPECTS

BC is the most common malignant tumor among women, marked by high clinical, morphological, and biological heterogeneity^[3]^. According to the ACS, BC is the second leading cause of cancer death in women, second only to lung cancer. Its incidence has increased by around 1% per year in recent years, with an estimated 313,510 new cases and 42,780 deaths in 2024^[1]^. The multifactorial nature of BC involves cellular modifications influenced by the microenvironment and organic factors, which favor its formation, progression, and invasion. Major risk factors include genetic alterations, such as mutations in the *BRCA1*, *BRCA2*, *TP53*, *PTEN*, and *CDH1* genes^[16,17]^; age (particularly for women over 50); family history; menopause; hormone replacement therapy; obesity; and environmental factors such as alcohol consumption, smoking, and drug use^[2,18]^.

Perou *et al*. (2000) were the first to demonstrate that, despite phenotypic diversity, BC can be grouped based on intrinsic gene expression patterns^[19]^. This study identified five main subtypes of BC: luminal A, luminal B, HER2 overexpression, basal-like breast cancer (BLBC), and normal-breast-like tumors^[19]^. Immunophenotypic classification by hormone receptors (estrogen receptor (ER) and progesterone receptor (PR), HER2, and the cell proliferation index-Ki-67 is widely used in clinical practice due to the complexity and cost of gene expression profiling^[20]^. The St. Gallen International Breast Cancer Conference (2013) classified BC into molecular subtypes based on these parameters: luminal A (ER+, PR+, HER2-, Ki-67 < 20%), luminal B (ER+, PR+ < 20%, HER2-, Ki-67 ≥ 20%), HER2- enriched (ER-, PR-, HER2+), and basal-like (ER-, PR-, HER2-)^[21]^.

Luminal A and B subtypes present high expression of genes typical of the luminal epithelium of the breast, such as *ESR1*, *GATA3*, *FOXA1*, *XBP1*, *MYB*, and high frequency of *PIK3CA* mutation^[22]^. The Luminal A subtype, which represents 40% to 50% of BC cases, is generally low-grade and associated with a good prognosis. In contrast, the luminal B subtype tends to be a higher grade and has a worse prognosis compared to luminal A^[20]^. The HER2-overexpressing subtype (approximately 15% of invasive BC) presents a high-grade and aggressive clinical course but responds well to anti-HER2 therapies, which have shown satisfactory results^[20]^. The basal-like subtype, which expresses genes associated with basal myoepithelial cells of the breast (such as keratin 5, 6, and 17), is characterized by frequent mutations in *TP53*, loss of *RB1*, inactivation of *BRCA1*, mutation in *PIK3CA*, *ATM* mutation, high activation of *MYC*, amplification of cyclin E1 and high expression of genes associated with cell proliferation^[22]^. This subtype has a triple-negative phenotype, high grade, high proliferation index, and poor prognosis with recurrences within 5 years of diagnosis^[23]^.

TNBC is frequently associated with the basal-like subtype (BLBC), with an overlap of approximately 56% of gene expression profiles. The coincidence rate of profiles between TNBC and BLBC ranges from 60%-90%, indicating that a large proportion of triple-negative tumors present features of the basal-like subtype^[3]^. TNBC is the most aggressive subtype of BC, with a worse prognosis, and a high frequency among young women, especially those under 40 years old, corresponding to 10%-15% of BC cases^[24]^. Thus, we will consider that TNBC is a heterogeneous disease, with distinct molecular subtypes within the triple-negative classification, each with different responses to chemotherapy^[25]^. The six subtypes of TNBC include (i) basal-like 1; (ii) basal-like 2; (iii) immunomodulatory; (iv) mesenchymal; (v) mesenchymal stem-like; and (vi) luminal androgen receptor^[25,26]^. The mortality rate in patients with TNBC is high, reaching up to 40%, with a median survival time of 13.3 months and a high propensity to metastases to the brain and visceral organs. Thus, TNBC is widely recognized as a highly aggressive, invasive, poorly differentiated form of BC and strongly associated with metastasis and EMT^[3]^.

### Metastasis and EMT

Metastasis represents one of the most challenging conditions for patients with BC and represents a critical factor in the failure of oncological therapies^[27]^. This process involves the progression of the primary tumor to distant secondary tumors and encompasses cellular mechanisms, such as dissociation from the primary tumor, invasion, immune evasion, and tissue microenvironment regulation^[28]^. Metastatic spread, a potentially lethal trait, profoundly affects prognosis and impacts about 30% of BC patients, with common metastatic sites including the skeletal system, lungs, liver, and brain, sometimes occurring even years after initial recovery. Notably, around 75% of BC metastases involve bone, with a five-year survival rate of 22.8%, while lung metastasis carries a five-year survival rate of 16.8%^[27]^. Brain metastases develop in roughly 15%-30% of metastatic BC cases, significantly affecting quality of life and yielding a poor prognosis^[27]^.

Metastasis begins when transformed epithelial cells, typically bound tightly to each other and the extracellular matrix, undergo reprogramming to adopt a mesenchymal phenotype. This process is known as the EMT^[28]^. Various pathways and genes play essential roles in EMT, including integrins, *SRC*, *RAS*, Notch, and *Wnt/β-catenin*, with E-cadherin acting as a central molecule in this process^[29]^. E-cadherin is a transmembrane glycoprotein essential for adhesion between epithelial cells, and its loss facilitates invasion by cancer cells^[30]^. Reduced E-cadherin levels upregulate growth factors like TGF-β, reactive oxygen species, and apoptosis signaling pathway^[31]^. Additionally, E-cadherin inhibition by transcription factors such as TWIST1, SLUG, SNAIL, ZEB1, ZEB2, and FOX contributes to cancer initiation, progression, invasion, metastasis, and treatment resistance^[29]^. Abnormal expression of ZEB1 has been reported in multiple human cancers, including liver, colon, lung, and BC^[32,33]^. Inflammatory cytokines, such as interleukin 6 (IL-6), also play key roles in BC metastasis by promoting STAT3 activation. IL-6/STAT3 signaling triggers EMT by regulating the estrogen receptor α, which is essential in promoting metastasis^[34]^.

The response of TNBC to hormonal or anti-HER2 therapies is limited, as there is no expression of estrogen and PR, in addition to low levels of HER2 expression^[35]^. The standard TNBC treatment remains neoadjuvant chemotherapy followed by surgery in advanced cases; however, responses are often short-lived, with rapid relapses and higher rates of visceral and brain metastases^[35,36]^. Treatment of TNBC is complicated by the absence of specific molecular targets, which limits the efficacy of targeted therapies^[35]^. Novel therapeutic approaches are under investigation, including immunotherapies, tumor stromal targeting, modulation of DNA damage response, intracellular or surface receptors, signaling pathway inhibitors, and cell surface markers for drug delivery, such as antibody-drug conjugates^[24]^.

### Role of miRNAs in TNBC

Hormonal therapies and monoclonal antibodies are generally ineffective in treating TNBC, and some patients experience poor outcomes even after chemotherapy^[7]^. Identifying novel molecular targets is critical to improving prognosis and developing specific and effective therapies for TNBC patients^[7]^. Among the potential molecular targets, miRNAs are promising targets. These small, non-coding RNAs (18-24 nucleotides) specifically regulate targets, and studies indicate they play important roles in cellular processes, including cell survival, apoptosis, differentiation, aging, carcinogenesis, and metastasis^[13]^. The first association of miRNAs with cancer was reported by Calin *et al*. (2004) in chronic lymphocytic leukemia when they identified two miRNAs (miR-15a and miR16-a) in the absence of a tumor suppressor gene on chromosome 13^[37]^. Currently, miRNAs have been studied in several types of cancer, including gastric, colorectal, cervical, ovarian, prostate, bladder, and breast. In TNBC, several miRNAs modulate gene expression through transcriptional and epigenetic mechanisms, influencing tumor progression^[38]^.

A variety of miRNAs, such as miR-9, miR-21, miR-93, miR181a/b, miR-182, miR-221, and miR-155, have been shown to influence carcinogenesis in TNBC by coordinating and regulating these processes^[39]^. For instance, miR-9 has oncogenic functions in TNBC by binding to E-cadherin and reducing its expression, activating the β-catenin signaling pathway, and triggering cell motility and invasion^[40]^. Additionally, miR-181a, which is positively regulated by TGF-β and overexpressed in TNBC, promotes invasion and metastasis^[41]^. Inhibiting miR-181a may block EMT, invasion, and migration mediated by TGF-β, suggesting its anti-metastatic potential and therapeutic relevance^[42]^. Furthermore, Liu *et al*. (2015) identified four other miRNAs- miR-374b-5p, miR-218-5p, miR-126-3p, and miR-27b-3p- as potential prognostic indicators in TNBC^[43]^.

The miRNA-200 family (miR-200a, miR-200b, and miR-200c) is a tumor suppressor, especially in the regulation of EMT in metastatic TNBC^[7]^. miR-200a inhibits EMT by suppressing ZEB1 and ZEB2, and promoting E-cadherin expression, maintaining the epithelial phenotype^[44]^. miR-200b is a major EMT and metastasis regulator that blocks these processes by inhibiting *Kindlin-2* expression, migration, and metastasis through *PKC α* and *FUT4* suppression, as well as by inactivating EGFR and PI3K/AKT/mTOR signaling in TNBC cell lines like MDA-MB-231 and MCF-7^[45,46]^. miR-200c also promotes the retention of the epithelial phenotype, suppresses cell proliferation, and induces apoptosis in TNBC, establishing the miRNA-200 family as a valuable therapeutic target^[47]^.

Another notable miRNA is miRNA-34a, known for its antitumor action in several types of cancer, including BC. miR-34a suppresses the PI3K/AKT/mTOR pathway, resulting in apoptosis and cell cycle arrest, limiting tumor growth^[48]^. *In vitro* experiments showed that overexpression of miR-34a suppressed migration and invasion in BC cells, whereas low expression of miR-34a in 173 TNBC patients showed worse survival rates^[49]^. The synergistic activity of miR-34a with other tumor-suppressive molecules and its impact on the PI3K/AKT pathway have made it an attractive target for miRNA-specific therapies and prognostic biomarkers in BC^[50]^. In addition to the miRNAs already mentioned, others are also relevant in TNBC, standing out as potential therapeutic targets. miR-146a acts in the modulation of cell invasion and metastasis, regulating genes associated with the inflammatory and immune response in the tumor microenvironment (TME)^[51]^. Among the miRNAs with critical roles in TNBC, miR-10b, miR-145, and miR-29 are key regulators linked to processes such as EMT and cancer stem cell properties, both of which are associated with tumor invasiveness and metastatic potential, underscoring their potential value in prognostication and therapy^[52]^. Thus, these miRNAs broaden the research perspectives in TNBC, favoring therapeutic approaches that consider everything from tumor invasion to the influence of the microenvironment on the development of the disease.

### Therapeutic potential of miRNAs

Research on miRNAs is emerging as a promising, innovative approach for developing precise, minimally invasive diagnostic tools and potential therapeutic targets, especially in BC^[7]^. RNA therapeutics offer several advantages over protein-based approaches, including programmability, temporary activity within endogenous cellular pathways, easy manipulation, and non-immunogenicity^[53]^. Direct miRNA exposure to BC cells remains ineffective for therapeutic purposes due to miRNA susceptibility to nuclease degradation, rapid clearance, immunotoxicity, and low tissue permeability^[14]^. Consequently, efforts have been focused on miRNA delivery systems for circulation stability, including viral vector-based systems, chemically synthesized nanoparticles, and EVs as miRNA vehicles^[54]^.

## EVs AS THERAPEUTIC MESSENGERS

Heusermann *et al*. showed that the internalization of EVs is more efficient than that of synthetic nanocarriers, which tend to accumulate on the cell surface, while EVs are rapidly internalized without this accumulation^[55]^. Due to their synthetic nature, nanocarriers are often not recognized by the organism, which can generate adverse immune responses and rapid elimination by the immune system, reducing their effectiveness in the delivery of therapeutic mRNAs. This can lead to limited accumulation in the target tissue and suboptimal delivery of the therapeutic content^[56]^. Given these limitations, EVs emerge as a more effective solution, as they are naturally recognized by the organism and have biochemical characteristics that allow a favorable and efficient biodistribution for the delivery of mRNAs and other genetic materials^[57]^.

The release of EVs by cells was first interpreted as a waste disposal mechanism, facilitating the elimination of unnecessary compounds and playing roles in mineralization and platelet processes, hence the term “platelet dust”^[58]^. Subsequent studies expanded this view by demonstrating that EVs play essential roles in the regulation of the immune system^[59]^, and in the transfer of proteins and RNA between cells, acting as intercellular vehicles of communication and modulation^[60,61]^. Currently, EVs are understood as essential mediators of cellular component exchange and signaling vehicles in both homeostatic and pathological processes^[62]^. They consist of a heterogeneous class of lipid bilayer-enclosed particles released by eukaryote cells, archaea, and bacteria^[63]^. EVs can be classified by subpopulations based on biogenesis and size: exosomes (50-150 nm) originate from endosomal membrane budding; microvesicles (50-1,000 nm) from external membrane budding, and apoptotic bodies (> 1,000 nm), which arise during cell death^[64]^. Their composition, depending on the cell origin and environmental conditions, includes lipids, carbohydrates, proteins, and nucleic acids^[65]^. EVs can be isolated from cell cultures and biological fluids like blood, urine, and cerebrospinal fluid^[66]^.

### EVs derived from MSCs

MSCs, first described by Friedenstein *et al*. (1970)^[67]^, are emerging as a promising tool in cellular therapy due to their characteristics of self-renewal, high expansion potential, low immunogenicity, and secretion of tissue regeneration mediators^[68]^. They are easily obtained and can be isolated from various tissues, such as peripheral blood^[69]^, umbilical cord^[70]^, and adipose tissue^[71]^. The paracrine mediators of MSCs are responsible for their modulatory effects, and numerous studies have highlighted the importance of EV release as regulatory agents in these therapeutic effects^[8]^. Mesenchymal stem cell-derived EVs (MSC-derived EVs) can transfer proteins, mRNAs/miRNAs, or DNA molecules between cells via paracrine or endocrine signaling, offering a channel for biological modulators^[72]^. EV-based MSC treatments are considered safer due to their inability to directly form tumors and contain lower amounts of membrane proteins such as MHC molecules^[73]^. This aspect is especially relevant for a potential therapeutic application, given that tumor EVs, in contrast, are often associated with the promotion of metastatic environments and can be associated with relapse and metastasis in cancer patients^[74,75]^. Therefore, MSC-derived EVs are being studied as new intercellular communication frontiers, regulating cellular behaviors like immune modulation, angiogenesis, proliferation, and migration, as well as showing therapeutic potential for various clinical diseases, especially BC^[72]^.

### Therapeutic potential of MSC-derived EVs

The complexity of the interaction between tumor cells and resident cells makes EVs important factors in intercellular communication within the TME^[76]^. Chemotherapy, radiotherapy, and surgery are the current treatments for TNBC; however, improving therapeutic efficacy and reducing drug side effects remain the main challenges^[4]^. Currently, therapy based on MSC-derived EVs has been studied due to their great potential in regenerative medicine and their unique advantages^[77,78]^. MSC-derived EVs contain pluripotent and signaling molecules such as cytokines, miRNAs, and growth factors that promote tissue repair and regeneration; exhibit low immunogenicity; have immunoregulatory functions capable of modulating immune cell functions and promoting an anti-inflammatory environment; deliver specific genes and proteins to target cells; and ensure safety because they lack self-replication abilities, minimizing the risk of tumor formation^[78]^. The characteristics of the TME can be altered by MSC-derived EVs through the regulation of immune cells, fibroblasts, and vascular endothelial cells, promoting an unfavorable environment for tumor growth and dissemination^[79]^. Furthermore, immunomodulators such as TGF-β and IL-10, which are present in their content, can inhibit inflammatory responses and immune escape mechanisms, helping to reduce inflammation in the TME and inhibit cancer progression^[80]^.

The content of proteins, mRNAs, miRNAs, and lipids in MSC-derived EVs is important for targeting tumor cells and regulating their behavior, showing an important role in TNBC cell proliferation^[81]^, metastasis^[82]^, immune evasion^[83]^, and angiogenesis^[84]^. MSCs-derived EVs can be used as carriers of therapeutic drugs and genes for TNBC treatment^[78]^. Co-delivery mediated by EVs loaded with the chemotherapeutic drug doxorubicin and cholesterol-modified miRNA-159 demonstrated synergistic efficacy *in vitro* an *in vivo* experiment using a xenograft mouse model^[85]^. Enhanced anticancer effects, such as tumor growth inhibition and increased survival rates, were observed, enabling effective tumor therapy^[85]^. Furthermore, MSC-derived EVs consisted of a favorable delivery system for harmful drugs such as vincristine sulfate (VCR), as demonstrated by Farouk *et al*. (2024) in BC treatment. VCR sulfate loading provided more effective targeting than the free drug, promoting targeted delivery and decreased side effects^[86]^.

The transfer of miRNAs from MSC-derived EVs to target cells is capable of regulating important PI3K/AKT and TLR4/NF-kB signaling pathways, as well as the secretion of cytokines IL-6, IL-10, TNF-α, and IL-1β^[87,88]^. Local administration of human placental MSC-derived EVs (hPmsMSC-EVs) in murine xenograft models derived from 4T1 lineage suppressed tumor cell proliferation and migration through indirect antiangiogenic mechanisms, corroborating previously obtained *in vitro* results^[89]^. These nanoparticles can migrate to injured, inflamed tissues and immune organs, acting as immune system suppressors and inhibiting antitumor immunity in BC *in vivo* through the upregulation and downregulation of PD-L1 on myeloid cells and T cells, respectively^[83]^. The study by Li *et al*. (2020) evaluates the therapeutic activity of EVs extracted from adipose tissue mesenchymal stem cells (ADSC-EVs) with high and low expression of CD90 in immunocompetent mouse models of TNBC. The results showed that ADSCs-EVs with low CD90 expression suppressed tumor growth by reducing proliferation and migration, while also promoting apoptosis in tumor cells. Furthermore, when ADSC-EVs with low CD90 expression were loaded with miRNA-16-5p, an increased antitumor effect was observed^[90]^. Despite the potential present in therapy with EVs derived from MSCs, there are still many challenges to be faced. Therefore, continued efforts in basic research, technological innovation, and clinical trials are needed for the application of this therapy in patients with TNBC^[78]^.

### Immunosuppression induced by MSC-derived EVs

The source of EV isolation is a critical determinant of their therapeutic effects in either tumor promotion or suppression. MSC-derived EVs have therapeutic effects similar to those of their parental cells, having dual modulatory effects, influencing both innate and adaptive immune response through interactions with T cells, B cells, dendritic cells (DC), natural killer cells (NK), and macrophages^[91]^. A promising source for EV isolation is umbilical cord mesenchymal stem cells (hUCMSC-EVs), due to their low tumor-promoting potential and lower immunogenicity, making them more suitable for use in allogeneic therapies^[92]^. MSC-derived EVs have anti-inflammatory properties and play a crucial role in modulating immune responses through the suppression of pro-inflammatory cytokines such as TNF-α and IL-1β, and the increase in anti-inflammatory mediators such as IL-10, IL-6, and TGF-β^[79]^. A notable example is their ability to reprogram macrophages into the M2 phenotype via miRNA transfer, such as miR-146a and miR-223, which regulate inflammatory pathways^[93]^. The study by Li *et al*. (2019) portrays the ability of MSC-derived EVs to polarize M2 macrophages through activation of the miR-let7/HMGA2/NF-κB pathway^[94]^. The MSC-derived EVs cargo may present immunomodulatory proteins and enzymes such as indoleamine 2,3-dioxygenase (IDO), prostaglandin E2 (PGE2), and galectins capable of suppressing the proliferation and activation of T cells^[95,96]^, as well as expanding regulatory T cells (Treg) while inhibiting TH17 cells to promote immune homeostasis^[79]^. Zhou *et al*. (2021) showed that BMMSC-EVs loaded with galectin-9 siRNA and oxaliplatin (iEXO-OXA) promoted antitumor immunity through macrophage polarization, cytotoxic T lymphocyte recruitment, and Treg downregulation in cancer treatment^[97]^.

The immunosuppression exhibited by MSC-derived EVs may be a way to prevent an exacerbated inflammatory response, in order to protect the TME. In contrast, bioengineered MSC-derived EVs loaded with substances are capable of stimulating an antitumor response^[91]^. A study involving renal cell carcinoma showed that the administration of hUCMSC-EVs loaded with miR-182 promoted the proliferation of T and NK cells with suppression of tumor growth and metastatic potential^[98]^. In turn, Fan *et al*. (2019) observed the inhibition of the proliferation and function of NK cells by MSC-derived EVs through the mechanism of positive regulation of TGF-β in the downstream TGF/Smad2/3 signaling pathway^[99]^. The activation of Toll-like receptor (TLR) signaling, together with Treg cells by EVs derived from human embryonic stem cells, promoted attenuation of the immune response^[100]^. Likewise, hUCMSC-derived EVs bound to blood monocytes of M2 macrophage origin and produced immunosuppressive cytokines^[101]^.

The use of EVs as delivery vehicles for miRNAs has direct implications in the immunological context of BC, particularly regarding resistance to immune checkpoint inhibitors (ICIs)^[102]^ and CD8+ T cell exhaustion^[103]^. The TME often induces resistance to ICIs through complex interactions between tumor cells and immune cells, creating an immunosuppressive state. In this scenario, EVs can modulate the expression of miRNAs that influence both the adaptive immune response, by regulating T cell functionality and tumor resistance^[102]^. In the case of exhausted CD8+ T cells, the presence of miRNAs in EVs could restore their function, reversing cellular exhaustion and enhancing the immune response against the tumor^[103]^. These findings open new possibilities for combined therapies that integrate EVs as therapeutic vehicles to reverse immunosuppression and improve the efficacy of immunological therapies, such as ICIs, in BC treatment. The controversial immunological responses caused by MSC-derived EVs, such as tumor promotion and suppression, can be attributed to the complexity of the TME, tumor malignancy, and the systemic environment of the host. This highlights the need for further research in the area, mainly in the development of standards for the isolation and purification of EVs in different MSC culture conditions^[91]^. Therefore, it is crucial to detail all the procedures and parameters used, since they influence the content and production of the MSC-derived EVs^[91]^.

### Challenges of EV therapy

EVs offer a range of biological functions, from tissue homeostasis and inflammatory regulation to tumor growth and metastasis^[104]^. They can also interact with receptors on target cells, making them promising candidates for targeted delivery^[105]^. EVs are stable in circulation, biocompatible, and have low immunogenicity and toxicity, standing out as potential therapeutic vehicles for cancer treatment, tissue regeneration, and vaccines^[106,107]^. Transmembrane proteins anchored on the surface of EVs can increase endocytosis, delivering their contents to target cells^[108]^. Among these proteins, CD47 stands out as a transmembrane protein associated with integrins that protect cells from phagocytosis by binding to the signal regulatory protein alpha (SIRPα)^[109,110]^. Kamerkar *et al*. (2017) demonstrated that the presence of CD47 in exosomes contributes to the evasion of phagocytosis by circulating monocytes and increases their half-life in circulation^[111]^. Notably, EVs can efficiently cross biological barriers (e.g., tissue, cell, or intracellular compartments) and induce functional changes in target cells, overcoming the primary limitations faced by conventional synthetic delivery systems^[43,112,113]^.

Significant challenges exist with EV-based therapies, such as low yield and efficiency, necessitating a large number of cells to produce sufficient EVs for *in vitro* and *in vivo* use^[114]^. The amount of EVs secreted by cells is insufficient for clinical applications, as they generally yield less than 1 µg of EVs per 1 mL of culture medium^[64]^. Furthermore, there is a need for standardization of EV isolation methods that can ensure scalability and high purity for clinical use^[64]^. The ultracentrifugation method, while commonly used for EV isolation, often results in low yield and contamination with medium proteins. Additional methods such as filtration and size-exclusion chromatography are necessary to ensure the purity of the EVs for therapeutic use^[64]^. Another important factor is EV biodistribution, influenced by parameters like cellular origin, administration route, and EV composition. A systematic review by Kang *et al*.(2021) highlighted that EVs are distributed throughout the body, mainly in organs like the liver, lungs, kidneys, and spleen^[115]^. The biodistribution, stability, and therapeutic effect of EVs must be considered when selecting an administration route. The study by Li *et al*.(2018) is relevant for the development of large-scale therapies and biodistribution of cancer therapeutics, as it provides data on how anesthetics influence metastasis and angiogenesis^[116]^. Techniques such as bioluminescence and animal cancer models can be used to monitor the distribution of therapies at different stages of treatment, in addition to helping understand the impacts of anesthetics on the progression of TNBC^[116]^. Furthermore, the safe therapeutic dose range of EVs in humans needs further study^[72]^. Long-term research and clinical studies are necessary to advance knowledge of EV-based therapeutics.

## MIRNA AND EVs AS A THERAPEUTIC STRATEGY FOR BC

Research on miRNAs is advancing rapidly due to their potential as therapeutic agents and biomarkers. Key research areas include the use of EVs as delivery vehicles for miRNA, regulation of gene expression, and the development of combination therapeutic strategies^[12]^. Numerous studies have highlighted the role of miRNAs in EVs in TNBC tumorigenesis^[117,118]^, with a focus on diagnosis^[119,120]^, prognosis^[119,121]^, treatment selection^[122,123]^, and monitoring^[121]^.

The review by Berti *et al*. (2024) identified miRNAs in EVs, such as let-7a-5p and miR-155-5p, as promising biomarkers in liquid biopsies and prognosis in TNBC. Expression analyses in EVs derived from BC cell lines demonstrated upregulation of let-7a, miR-9, miR-10b, miR-328, miR-455, miR-602, miR-1246, miR-92b, miR-105, miR-122, miR-130a, miR146, miR149, miR155, miR188, and miR1255a and downregulation of miR-33, miR-130, and miR-198 for MDA-MB-231. The luminal A cell line (MCF-7) showed upregulation only for miR-198 and downregulation for Let-7a, miR-9, miR-10b, miR-92b, miR-130a, miR-146, miR-149, miR-155, miR-188, miR-328, miR-602, and miR-1246^[39]^. Some miRNAs have also been studied as modulators of EMT markers in BC. It was observed that miR-21-5p, miR-143-3p, or miR-378 carried by exosomes increased EMT markers in MDA-MB-231 cells^[124]^. Similarly, co-incubation of microvesicles derived from MDA-MB-231 cells and loaded with miR-221-3p promoted EMT in MCF-7 cells^[125]^.

EVs show promise as vehicles for targeted miRNA therapy to restore tumor-suppressor miRNAs or inhibit oncomiRs^[126]^. Systematically reviewed EV-based clinical trials, compiling 471 studies on EV-related therapies for diverse diseases; however, only 19.32% investigated therapeutic strategies, the vast majority of which were aimed at diagnosis. Of the therapeutic studies analyzed, 21.43% addressed EV loading therapy with therapeutic molecules, and only 11% were aimed at cancer^[126]^. Despite a wide range of studies on miRNAs in EVs in BC, few explore the targeted therapy of miRNAs of interest through EVs as delivery vehicles. Initial research indicates that exosomes from MSCs carrying miR-381^[127]^ and miR-218^[128]^ reduced the viability, migration, and invasion of TNBC cells (MDA-MB-231 cell line) by modulating the Wnt signaling pathway and the transcription factors Snail and Twist linked to the EMT process and promoting the expression of epithelial markers (CDH1). Exosomes from MSCs carrying miR-34a showed significant effects in reducing the migration and invasion of TNBC cells, as well as inducing apoptosis via downregulation of *Bcl2* and *c-MET* in a dose-dependent manner^[129]^.

Another approach involves the use of miR-424-5p-loaded exosomes as a delivery system for *in vitro* and *in vivo* TNBC treatment. EV-miR424 suppressed PD-L1 expression, facilitated an inflammatory microenvironment (enhancing IFN-γ, TNF-α, and IL-6 secretion while reducing IL-10), and promoted tumor cell apoptosis through caspase activation and extracellular lactate dehydrogenase release. *In vivo* studies further demonstrated tumor growth suppression, underscoring the therapeutic potential of miR-424-loaded EVs in TNBC^[130]^.

Exosomes derived from human umbilical cord cells are efficient nanocarriers of miR-3182 for TNBC cells. Khazaei-Poul *et al*. demonstrated the decrease in cell proliferation and invasion, as well as the induction of apoptosis through the negative regulation of the *mTOR* and *S6KB1* genes, important in the PI3K/AKT/mTOR signaling pathway^[131]^. Exosome-mediated administration of miR-145 in the T-47D cell line promoted decreased expression of genes such as *MMP9* and *ERBB2*, as well as increased gene expression of *TP53* and *ROCK1*, promoting an anticancer effect enhanced by miR-145^[132]^. O’Brien *et al*. (2018) enriched EVs secreted from MSCs with miR-379 and investigated their therapeutic potential in the HCC-1954 cell line. Their results showed that EVs-miRNA administration was tolerated *in vivo*, without adverse effects, and with a significant reduction in tumor growth during six weeks of monitoring^[82]^. In summary, while there is substantial research on miRNA analysis in EVs for BC, studies specifically exploring targeted miRNA delivery via EVs remain limited. However, these approaches show promise, with specific miRNAs demonstrating potential for reducing TNBC aggressiveness, making EVs a viable and targeted therapeutic strategy in future oncologic treatments [Table 1].

**Table 1:** Mesenchymal stem cell-derived EVs in miRNA-targeted therapy in breast cancer

**EVs- miRNAs**	**EVs donor cells**	**EV isolation**	**Loading method**	**Recipient cells**	**Main findings**	**Ref.**
miR-145	AT-MSC	Exocib kit (Cib Biotech)	Lentiviral transfection	T-47D	**↓** *MMP9, ERBB2* **↑** *TP53, ROCK1*	[132]
miR-218	AT-MSC	Exocib kit (Cib Biotech)	Electroporation method	MDA-MB-231	**↓** *Runx2, Rictor, CDH2,* viability, invasion, angiogenesis **↑** *CDH1*	[128]
miR-424	AT-MSC	Lipofectamine RNAiMAX reagent (Thermo Fisher Scientific)	Ultracentrifugation	HCC-1954; MDA-MB-231	**↓** *PDL-1*, *in vivo* tumor growth **↑** Apoptosis Induction of inflammatory microenvironment in MDA-MB-231	[130]
miR-3182	HUC-MSC	Exocib kit (Cib Biotech)	Electroporation method	MDA-MB-231	**↓** Proliferation, invasion, *S6KB1, mTOR* **↑** Apoptosis	[131]
miR-381	AT-MSC	Exocib kit (Cib Biotech)	Electroporation method	MDA-MB-231	**↓** Viability, migration, invasion, *CTNNB1, LRP6, Twist, Snail*	[127]
miR-34a	DP-MSC	ExoSpin Purification Kit (Cell Guidance)	Lentiviral transfection	MDA-MB-231	**↓** *Bcl2, c-Met*, proliferation, invasion, and migration **↑** Apoptosis	[129]
miR-379	MSC	Lentiviral transfection	Ultracentrifugation	HCC-1954	**↓** *in vivo* tumor growth Potent tumor suppressor in breast cancer	[82]

EVs: Extracellular vesicles; miRNAs: microRNAs; AT-MSCs: adipose tissue-mesenchymal stromal cells; HUCMSC: human umbilical cord mesenchymal stem cells; DP-MSCs: dental pulp-derived mesenchymal stromal cells; MSC: mesenchymal stem cells. **↑**: Increase; **↓**: decrease.

## CLINICAL TRIALS INVOLVING MSC-DERIVED EV THERAPY

**Figure 1:**
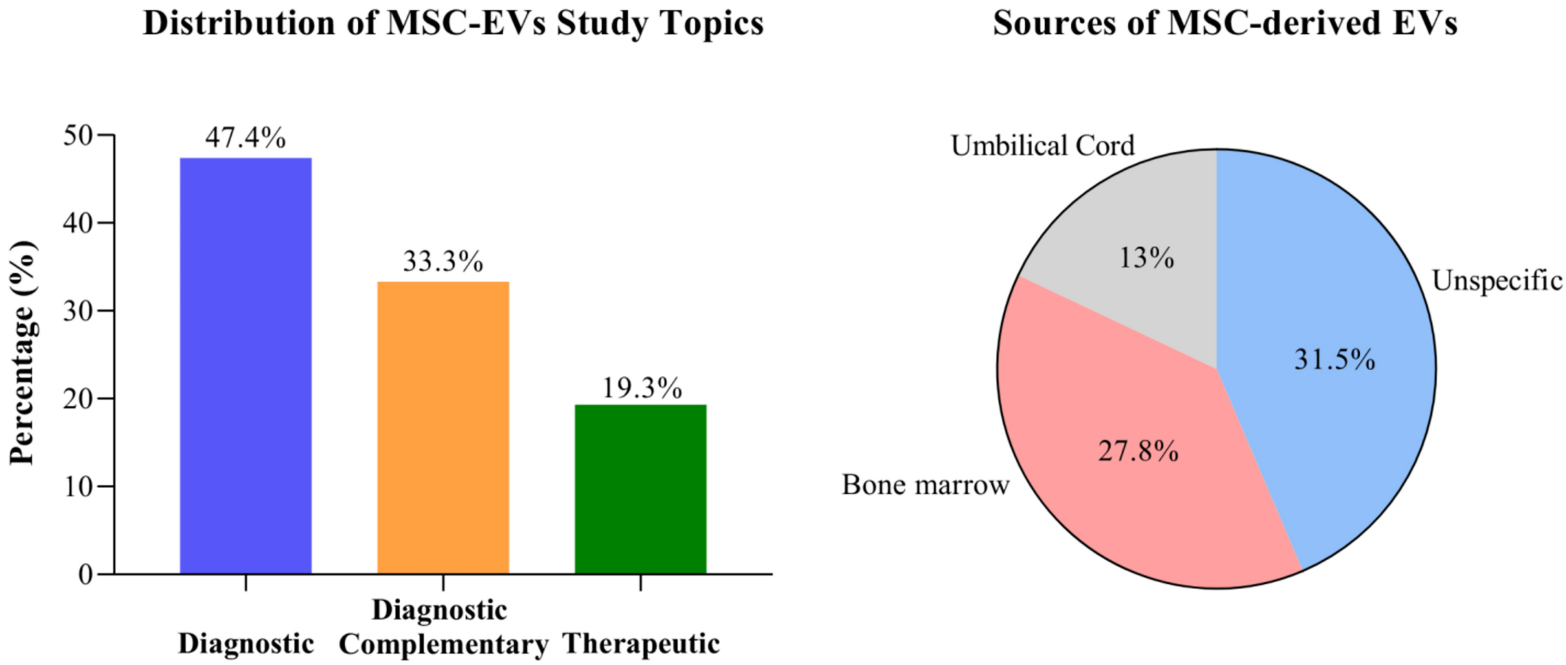
Quantitative breakdown of topics in reviewed studies on MSC-derived EV therapy. Quantitative analysis of the systematic review by Mizenko *et al*. (2024) involving clinical trials related to EVs and cited in this article^[126]^. Analysis of the distribution of prevalent topics in the studies, such as diagnosis, complementary diagnosis, and therapeutic, as well as the main sources of MSC-derived EVs used. Graph Pad Prism 8.0.2. MSCs: Mesenchymal stem cells; EVs: extracellular vesicles.

Kordelas *et al*. (2014) documented the first clinical trial of MSC-derived EVs in patients with graft-versus-host disease^[133]^. The study involved intravenous administration of MSC-derived EVs at escalating doses, given at 2- or 3-day intervals for two weeks. The treatment demonstrated a significant reduction in disease symptoms, without any reported side effects, and patients remained stable for more than four post-therapy^[133]^. A systematic review by Mizenko *et al*. (2024) included 471 clinical trials related to EVs in the ClinicalTrials.gov database, with publication dates up to December 2023^[126]^. Most studies presented EVs derived from MSCs (59.3%), originating from bone marrow (27.8%), umbilical cord (13%), or unspecified sources (31.5%). Most studies examined EVs for diagnostic purposes (47.4%), complementary diagnostics (33.3%), and only 19.3% related to therapeutics [Figure 1]. MSC-derived EV-based therapies were predominantly studied in respiratory diseases, such as COVID-19 and acute respiratory distress syndrome (ARDS)^[126]^. In cancer research, clinical trials primarily focused on diagnostics and complementary diagnostics, leveraging the ease of *in vitro* tumor cell growth and the innovative liquid biopsy approach for diagnosis. Only one therapeutic clinical trial involved using MSC-derived EVs for cancer treatment^[126]^. The clinical trial based on the studies of Kamerkar *et al*. (2024) and Tang *et al*. (2021) involves the use of KrasG120 siRNA (iExosomes) for the treatment of patients with pancreatic cancer harboring the KrasG120 mutation. This Phase 1 study is projected to conclude in April 2025 (identifier: NCT03608631)^[111,134]^. Current records from ClinicalTrials.gov (accessed in January 2025) showed two other studies involving MSC-derived EV therapy for rectal cancer (identifier: NCT06536712) and acute myeloid leukemia (identifier: NCT06245746) [Table 2]. Both studies are Phase 1, interventional trials, and are not yet recruiting patients. These findings underscore the need for research groups to further investigate the therapeutic mechanisms of MSC-derived EVs, particularly in TNBC, to advance preclinical studies into clinical trials.

**Table 2:** Clinical trials based on MSC-derived EVs in disease therapy

**NCT number**	**Title**	**Year**	**Disease**	**EVs source**	**Adminis-tration route**	**Status**	**Phase**	**Ref.**
NCT038631	iExosomes in treating participants with metastatic Pancreas cancer with KrasG12D mutation	2018	Pancreatic cancer	Mesenchymal stromal cells	Intravenous	Recruiting	1	https://ClinicalTrials.gov/show/NCT03608631
NCT06536712	Effects of exosome administration in preventing early leakage in rectal cancer patients undergoing low anterior resection	2024	Rectal cancer	Human placenta mesenchymal cells	Intraperitoneal	Not yet recruiting	1	https://clinicaltrials.gov/study/NCT06536712
NCT06245746	UCMSC-Exo for chemotherapy-induced myelosuppression in acute myeloid leukemia	2025	Acute myeloid leukemia	Umbilical cord-derived mesenchymal cells	Intravenous	Not yet recruiting	1	https://clinicaltrials.gov/study/NCT06245746

## CONCLUSION

It is worth highlighting that therapies based on miRNAs embedded in EVs represent a promising advance in precision medicine, particularly in the treatment of TNBC and other complex diseases. Recent studies have shown that EVs, unlike other mRNA delivery systems, offer high biological compatibility, which reduces the risk of adverse immune responses and improves the bioavailability of therapeutic miRNAs at the target site^[57]^. EV-mediated delivery not only facilitates the transfer of miRNAs that regulate critical tumor processes, such as cell proliferation and invasion, but also helps modulate the TME, allowing safer and more effective therapeutic interventions^[135]^.

The prospects for this technology include the development of genetically modified or bioengineered EVs, which can be programmed to deliver personalized microRNA therapies in response to specific stimuli, such as pH or metabolism of the tumor tissue. This approach would open up new possibilities for combination treatments and targeted therapies, maximizing therapeutic efficiency and minimizing side effects. Furthermore, with the expansion of research and clinical trials around EV-based therapies, the expectation is that this technology will advance beyond the laboratory, reaching clinical application for several types of cancer, including TNBC, and other diseases where therapeutic options are still limited. Therefore, EVs offer innovative therapeutic potential for the delivery of miRNAs, with major implications for the future of gene therapy and personalized treatment.

## DECLARATIONS

### Copyright

© The Author(s) 2025.
